# Imputation accuracy to whole-genome sequence in Nellore cattle

**DOI:** 10.1186/s12711-021-00622-5

**Published:** 2021-03-12

**Authors:** Gerardo A. Fernandes Júnior, Roberto Carvalheiro, Henrique N. de Oliveira, Mehdi Sargolzaei, Roy Costilla, Ricardo V. Ventura, Larissa F. S. Fonseca, Haroldo H. R. Neves, Ben J. Hayes, Lucia G. de Albuquerque

**Affiliations:** 1 School of Agricultural and Veterinarian Sciences, UNESP, Jaboticabal, SP 14884-900 Brazil; 2 National Council for Scientific and Technological Development, CNPq, Brasília, DF 71605-001 Brazil; 3 Ontario Veterinary College, UG, Guelph, Canada; 4 Select Sires Inc., Plain City, OH USA; 5 Queensland Alliance for Agriculture and Food Innovation, UQ, Brisbane, QLD 4072 Australia; 6 School of Veterinary Medicine and Animal Science, USP, Pirassununga, SP 13635-900 Brazil; 7 GenSys Associated Consultants, Porto Alegre, RS 90680-000 Brazil

## Abstract

**Background:**

A cost-effective strategy to explore the complete DNA sequence in animals for genetic evaluation purposes is to sequence key ancestors of a population, followed by imputation mechanisms to infer marker genotypes that were not originally reported in a target population of animals genotyped with single nucleotide polymorphism (SNP) panels. The feasibility of this process relies on the accuracy of the genotype imputation in that population, particularly for potential causal mutations which may be at low frequency and either within genes or regulatory regions. The objective of the present study was to investigate the imputation accuracy to the sequence level in a Nellore beef cattle population, including that for variants in annotation classes which are more likely to be functional.

**Methods:**

Information of 151 key sequenced Nellore sires were used to assess the imputation accuracy from bovine HD BeadChip SNP (~ 777 k) to whole-genome sequence. The choice of the sires aimed at optimizing the imputation accuracy of a genotypic database, comprised of about 10,000 genotyped Nellore animals. Genotype imputation was performed using two computational approaches: FImpute3 and Minimac4 (after using Eagle for phasing). The accuracy of the imputation was evaluated using a fivefold cross-validation scheme and measured by the squared correlation between observed and imputed genotypes, calculated by individual and by SNP. SNPs were classified into a range of annotations, and the accuracy of imputation within each annotation classification was also evaluated.

**Results:**

High average imputation accuracies per animal were achieved using both FImpute3 (0.94) and Minimac4 (0.95). On average, common variants (minor allele frequency (MAF) > 0.03) were more accurately imputed by Minimac4 and low-frequency variants (MAF ≤ 0.03) were more accurately imputed by FImpute3. The inherent Minimac4 Rsq imputation quality statistic appears to be a good indicator of the empirical Minimac4 imputation accuracy. Both software provided high average SNP-wise imputation accuracy for all classes of biological annotations.

**Conclusions:**

Our results indicate that imputation to whole-genome sequence is feasible in Nellore beef cattle since high imputation accuracies per individual are expected. SNP-wise imputation accuracy is software-dependent, especially for rare variants. The accuracy of imputation appears to be relatively independent of annotation classification.

**Supplementary Information:**

The online version contains supplementary material available at 10.1186/s12711-021-00622-5.

## Background

Compared to the use of genotypes from single nucleotide polymorphism (SNP) panels, genotypic information from whole-genome sequencing may improve prediction accuracies of breeding values for economically relevant traits since it potentially includes causal mutations for all phenotypes [1–3]. In spite of the drastic reduction in genome sequencing costs that has occurred in the last years, it is still cheaper to genotype rather than sequence the animals. An alternative and cost-effective strategy for obtaining sequence information for many animals is to sequence a small proportion of the population, and use it as reference to impute sequence data of animals genotyped with array technology [4].

Besides the potential benefit of improving the prediction accuracy of breeding values, sequence information can improve quantitative trait loci (QTL) mapping in genome-wide association studies [3, 5]. However, the benefits depend on how accurate is the sequence imputation process. Whereas the use of variants that are imputed with low accuracy can lead to obviously biased estimates, a more precise QTL mapping could be achieved with highly accurate sequence imputation [5, 6]. Accuracy of sequence imputation has been mainly assessed in single or multi-breed dairy cattle populations [5, 7–9]. Studies in this field are lacking for *Bos indicus* populations. Compared to taurine breeds, *B. indicus* presents, in general, lower levels of linkage disequilibrium (LD) between genetic markers at short distances [10] and a historically larger effective population size [11], which could make imputation more difficult.

In Brazil, Nellore (*Bos indicus*) is the predominant breed used for beef production, and various Nellore breeding programs started independently and have created reference populations based on high-density SNP arrays [12]. Using sequence data of influential Nellore bulls, which may allow the identification of about 36 million SNPs [13], all these reference databases can be imputed to the segregating DNA sequence. With the use of whole-genome sequence variants in genomic selection, the persistence of prediction accuracy can be maintained over several generations due to high LD of SNPs with causative mutations [1]. It is worth mentioning that increases in prediction accuracy with sequencing data have been achieved by adding preselected sequenced variants using GWAS to a regular SNP array [2, 14, 15]. However, in general, genomic prediction accuracies using all sequenced variants have been similar or slightly lower than those based on traditional SNP arrays [16–18].

The objective of the present study was to investigate the imputation accuracy to the sequence level in a Nellore beef cattle population, to verify the feasibility of the imputation process in this breed, which could contribute to defining the best strategy to impute sequence data to the existing sets of animals that were originally genotyped using commercial marker panels. Two imputation software, FImpute3 [19] and Minimac4 [20], were compared. FImpute3 uses family and/or population-based algorithms to infer and phase haplotypes and impute missing genotypes. Minimac4 is a population-based method that uses previously phased haplotypes, e.g. using the Eagle software [21], as input in both reference and target populations. We also investigated the accuracy of imputation of different functional annotation classes, with the hypothesis that functional variants may be more difficult to impute, as they might be more recent mutations (not yet removed by selection) and therefore in lower LD with array SNPs.

## Methods

### Whole-genome sequencing dataset

In total, 151 influential Nellore sires were chosen with the aim to optimize the imputation accuracy of our genotype database, comprised of about 10,000 Nellore animals genotyped with medium- (~ 35 k) to high-density (~ 777 k) SNP panels. For this, a k-means cluster analysis was performed using the genomic relationship matrix of the genotyped animals. The number of clusters was set equal to 151 and, within each cluster, the sire with the largest number of genotyped progenies was chosen for sequencing. A genomic relationship representation of the sequenced and genotyped animals is provided in [Additional file 1: Figure S1] with a PCA plot of the genomic relationship matrix.

The whole-genome sequencing of the sires was performed using the Illumina sequencing-by-synthesis technology at an overall average sequence coverage, after quality control (described below), of 14.5×, ranging from 7.8 to 26.3×. Fifty-two animals were sequenced using the Illumina HiSeq X™ Ten platform and 99 animals were sequenced using the Illumina NovaSeq™ platform.

### Variant calling and genotype quality control

Variant calling procedures were carried out following the guidelines provided by the 1000 bull Genomes Project, available at http://www.1000bullgenomes.com/doco/1000bullsGATK3.8pipelineSpecifications_Run8_Revision_20191101.docx. Both SNPs and insertion/deletion mutations were identified, but only SNPs were considered for this imputation study. After generating a variant call format file containing SNP information for each of the 151 sires, a quality control filtering step was implemented, using the VariantFiltration tool from the GATKv3.8 software [22], using the exclusion criteria suggested by [23]: quality by depth—QD < 2.0; Fisher Strand test—FS > 60.0; root mean square of the mapping quality score—MQ < 40.0; ranked sum test for the distance of alleles from the end of the reads—ReadPosRankSum <  − 8.0; mapping qualities of reads—MQRankSum <  − 12.5; and SOR > 3.0. Next, the VCFtools software [24] was used to exclude non-biallelic markers and also those with a minor allele frequency lower than 0.01. Marker genotypes with a phred-scaled confidence (a genotype quality score) less than 15 were treated as missing and those SNPs with missing values for more than 40 individuals (26.5% of the total population) were removed from the analyses. After genotype quality control, 30,394,484 SNPs located on autosomes remained. As 150 of the sequenced sires had also been genotyped with the Illumina BovineHD Beadchip (~ 777 K), we verified the rate of concordance between the genotypes obtained from the genotyping and from the sequencing, and found an average of 99.6% of genotype concordance, ranging from 97.3 to 99.9%.

### Assessment of imputation accuracy

Imputation for the sequence level variants was carried out using two software: FImpute v3 [19] and Minimac4 [20]. FImpute3 was run considering only the population-based algorithm, which uses a deterministic approach to phase the haplotypes and to impute all the missing genotypes. It is worth mentioning that we have also run FImpute3 including the pedigree information and the results (not shown) were quite similar to those without pedigree. For Minimac4, reference and validation datasets were phased, separately, using the Eagle v2.4.1 software [21]. In contrast to FImpute, both Eagle and Minimac require reference and validation datasets split by chromosome. Also, in order to be more computationally efficient than its older versions, Minimac4 requires reference panels in M3VCF format, which were obtained using Minimac3 (see https://genome.sph.umich.edu/wiki/Minimac4). FImpute, Eagle and Minimac were run with default parameters in an Intel® Xeon® server with 1 TB of RAM memory and 72-core processors running at 2.70 GHz. To evaluate the software processing time efficiency in a similar multi-core system, each software was run parallelizing the 29 chromosomes in 58 processors (2 processors per chromosome).

The accuracy of imputation was investigated using a fivefold cross-validation scheme. The 151 animals with sequence information were randomly divided into five groups. Thus, five imputations were performed in such a way that a different group (target population) has all their genotypes masked except those that overlapped with the high-density (HD) SNP panel (~ 777 K). Table 1 shows the distribution of variants per chromosome.

**Table 1:** Distribution of SNPs by chromosome

Chr	Length (Mb)	Number of SNPs reference	Number of SNPs target	%SNPs to be imputed
1	158.44	1,965,500	36,424	98.15
2	136.15	1,594,559	30,832	98.07
3	121.00	1,396,284	28,583	97.95
4	119.86	1,514,666	26,434	98.25
5	120.05	1,350,367	25,150	98.14
6	117.80	1,461,356	28,480	98.05
7	110.64	1,297,470	25,926	98.00
8	113.24	1,309,174	26,848	97.95
9	104.64	1,281,310	25,088	98.04
10	103.26	1,219,761	22,454	98.16
11	106.98	1,246,742	24,204	98.06
12	87.20	1,121,539	19,004	98.31
13	83.45	947,897	17,794	98.12
14	82.37	991,359	20,322	97.95
15	84.96	1,120,179	18,849	98.32
16	80.98	974,221	18,626	98.09
17	73.15	928,571	17,803	98.08
18	65.81	753,566	14,541	98.07
19	63.42	713,820	13,370	98.13
20	71.96	910,476	16,074	98.23
21	69.84	857,266	16,234	98.11
22	60.76	729,871	13,479	98.15
23	52.50	774,175	12,292	98.41
24	62.30	790,397	14,192	98.20
25	42.34	521,876	9,425	98.19
26	51.98	655,622	11,869	98.19
27	45.61	642,015	10,030	98.44
28	45.91	636,577	9,991	98.43
29	51.09	687,868	10,717	98.44
Overall	2487.69	30,394,484	565,035	98.14

Number of SNPs reference: the overall and per chromosome number of SNPs present in the reference animals; Number of SNPs target: the overall and per chromosome number of SNPs present in the validation (target) animals

The squared Pearson’s correlation between observed and imputed genotypes (${R}^{2}$) and the percentage of correctly imputed genotypes (PERC), averaged across the fivefold cross-validation, were used to assess imputation accuracy. Both statistics, calculated by individual and by SNP, were computed only for the imputed SNPs (29,829,449 SNPs). In addition, we evaluated the relationship between the empirical imputation accuracies and the Minimac4 (Rsq) statistic, which represents the squared correlation between imputed genotypes and true unobserved genotypes (https://genome.sph.umich.edu/wiki/Minimac3_Info_File). According to [25], the Minimac Rsq corresponds to an estimate of the imputation accuracy.

### SNP-wise imputation accuracy by minor allele frequency class and functional annotation

The minor allele frequency (MAF) was computed by using Plink v1.9 [26]. Ensembl variant effect predictor (VEP) [27] was used to annotate all the SNPs to their functional effect. For each variant, the VEP identifies all the overlapping transcripts and then predicts the effects that each allele of the variant may have on each transcript. Variants were classified according to their functional impact in proteins as follows: (1) high: variants that cause premature stop codons, loss of function or trigger nonsense-mediated decay; (2) moderate: non-disruptive variants that might change protein effectiveness; (3) low: variants mostly harmless or unlikely to change protein behavior; and (4) modifier: non-coding variants or variants that affect non-coding genes, for which predictions are difficult or there is no evidence of impact.

## Results and discussion

Imputation accuracies per animal were high and consistent across methods and statistics (Table 2). The average (minimum and maximum) for the ${R}^{2}$ and PERC statistics were, respectively, 0.94 (0.89 to 0.97) and 96.57 (93.97 to 98.55) using FImpute3 and 0.95 (0.91 to 0.98) and 97.14 (94.97 to 98.88) using Minimac4. These results are in line with the literature since moderate to high whole-genome sequence imputation accuracies have been reported for different populations. Defining the accuracy of imputation as the correlation (r) between observed and imputed genotypes, Van Binsbergen et al. [9] reported mean accuracies from the BovineHD panel per individual of 0.93, 0.94, and 0.95, depending on the scenario, in Holstein Friesian cattle; values from 0.90 to 0.95 were found for Fleckvieh and Holstein cattle [7]; and accuracies up to 0.97 were reported for sheep [25].

**Table2:** Imputation accuracy per animal from the Bovine HD BeadChip (~ 777 K) to whole-genome sequence in Nellore cattle using two imputation software and the average of fivefold cross-validation

	FImpute3	Minimac4
$${R}^{2}$$		
Mean (SD)	0.94 ± 0.014	0.95 ± 0.011
Min	0.89	0.91
Max	0.97	0.98
PERC		
Mean (SD)	96.57 ± 0.76	97.14 ± 0.66
Min	93.97	94.97
Max	98.56	98.88

${R}^{2}$, Squared Pearson’s correlation between observed and imputed genotypes; PERC, percentage of genotypes correctly imputed; SD, standard deviation; Min, minimum value; Max, maximum value

One of the main issues in imputing sequence from low- or even high-density SNP panels in any population is the huge number of SNPs that have to be imputed. The accuracy of imputation tends to decrease as the number of SNPs from the lower density SNP panel decreases. This leads to an increased distance between the SNP to be imputed and the nearest SNP on the lower density marker panel. In this sense, there is a consensus in the literature [9, 25] that imputation from a low-density SNP panel to the sequence level should be done using a stepwise strategy. First, imputation is performed from the lowest- to the next highest-density SNP panel, etc. and finally to the sequence. Since a previous study in Nellore cattle [28] had shown that an imputation accuracy higher than 0.97 could be achieved for imputations from a variety of low-density SNP panels, i.e. 15K, 20K, 50K, and 75K, to the high-density (~ 777 K), in this study, we focused only on the imputation accuracy from the BovineHD panel (~ 777 K) to the whole-genome sequence data.

Considering the total number of imputed variants (29,829,449 SNPs), the average imputation accuracy per SNP indicated by the PERC and ${R}^{2}$ statistics were, respectively, 96.5% and 0.85 using FImpute3 and 97.1% and 0.90 using Minimac4. It is important to mention that, unlike PERC that was computed for all the imputed genotypes, the ${R}^{2}$ statistic could not be calculated for the 159,153 SNPs and 665,854 SNPs in FImpute3 and Minimac4 results, respectively, due to the lack of variability of the imputed genotypes within SNPs. These variants are spread across the 29 autosomes and the majority of them (148,452 in FImpute3 and 420,681 in Minimac4) have an original MAF lower than or equal to 0.03, which suggests that FImpute3 is more sensitive in capturing the natural low variability of rare variants than Minimac4. To better investigate the differences in SNP-wise imputation accuracies between software, in the remaining analysis only the variants with an ${R}^{2}$ calculated for both FImpute3 and Minimac4 (29,115,307 SNPs) were used to compare the results. Considering these 29,115,307 markers, the average values of the PERC and ${R}^{2}$ statistics were, respectively, 96.7% and 0.86 using FImpute3, and 97.3% and 0.88 using Minimac4.

**Fig. 1:**
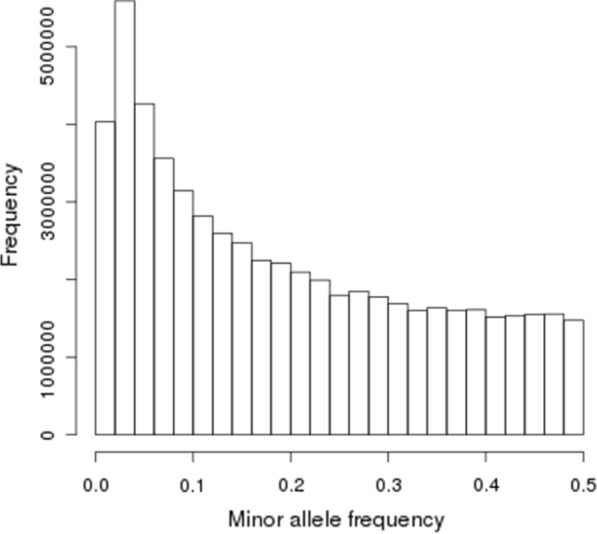
Minor allele frequency distribution in Nellore cattle at the sequence level

The imputation of rare variants is one of the most important issues that affect the average imputation accuracy in a specific population. This is especially true for whole-genome sequence imputation since it usually relies on imputing a high proportion of rare variants [8]. Here, the MAF distribution exhibited a high frequency of SNPs with a low MAF (Fig. 1), and the number of variants with a MAF ≤ 0.03 represented 13.5% of the total number. The average empirical accuracy of imputation by MAF [see Additional file 2: Figure S2] showed that, on the one hand, the lowest ${R}^{2}$ values were associated with the lowest MAF, and on the other hand, SNPs with a low MAF tended to show higher PERC. As stated by [29], PERC is a measure of how well genotypes are imputed whereas ${R}^{2}$ is a measure of how well the allele dosage is imputed. Thus, for low MAF variants, the concordance rate will be high because most genotypes are for reference homozygous animals, however it is very difficult to correctly impute alleles for animals that are heterozygous or alternate homozygous. Since ${R}^{2}$ is a statistic that is less allele-frequency dependent than PERC [30], henceforth, we will focus on the ${R}^{2}$ measure to evaluate imputation accuracy.

**Fig. 2:**
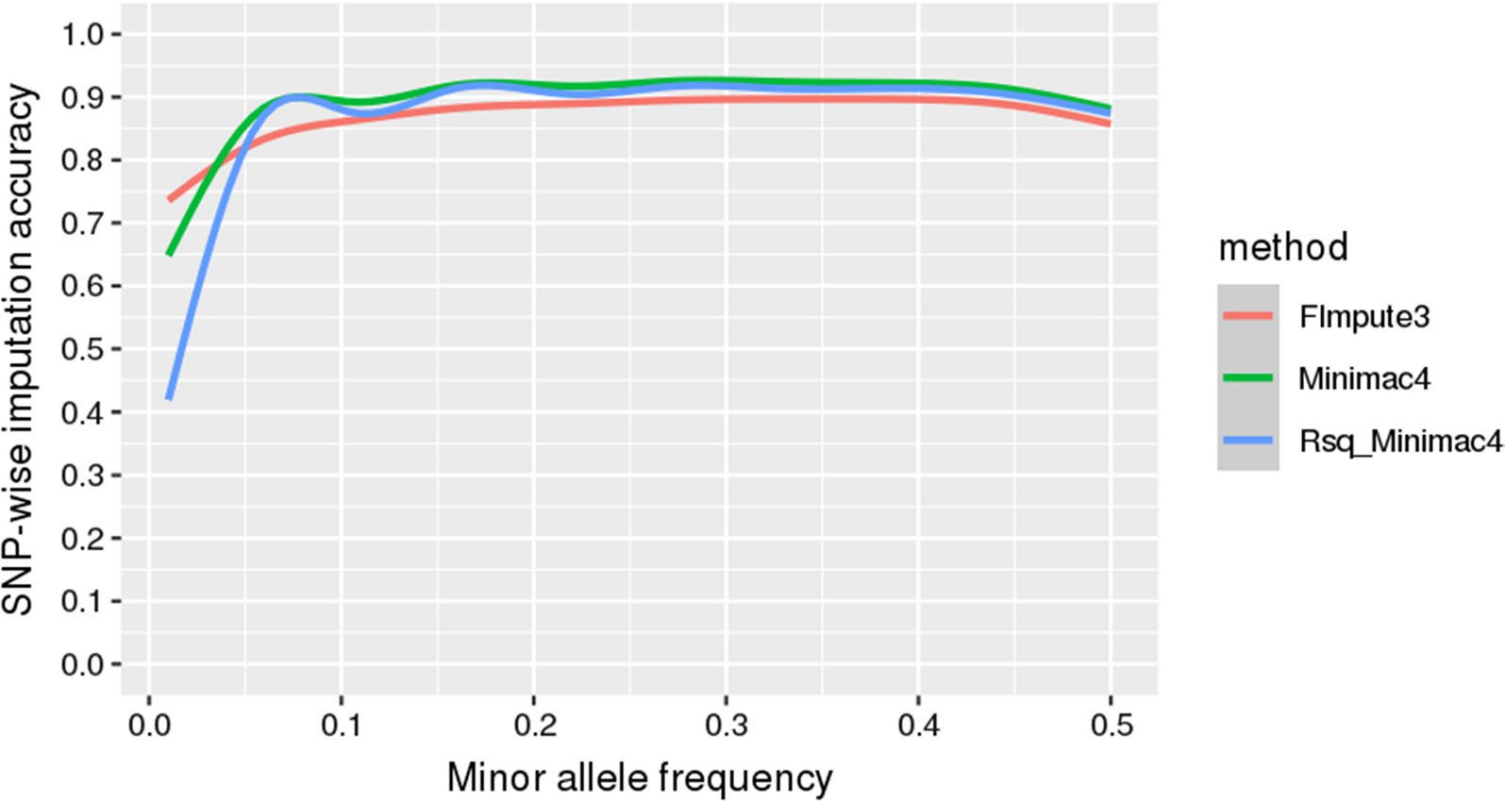
Smoothed conditional means (see http://search.r-project.org/library/ggplot2/html/geom_smooth.html) of the SNP-wise imputation accuracies by minor allele frequency (MAF). FImpute3 and Minimac4 methods correspond to the squared Pearson’s correlation between observed and imputed genotypes; and Rsq_Minimac4 method is an estimate of the squared correlation between imputed genotypes and true, unobserved genotypes (see https://genome.sph.umich.edu/wiki/Minimac3_Info_File)

Plotting the Minimac4 Rsq statistic together with the empirical imputation accuracy (${R}^{2}$) by MAF (Fig. 2) shows that the Minimac Rsq measure is a good indicator of the empirical imputation accuracy achieved by using the Minimac4 software, although it slightly underestimates accuracy across all MAF but more particularly for the lower MAF. This result concurs with that of Bolormaa et al. [25], who found that the Rsq Minimac statistic was a reasonable proxy of the empirical imputation accuracy in sheep. This statistic (Rsq) that reports the quality of imputation is a notable useful feature of Minimac and enables the filtering out of poorly imputed variants before any further analysis [25, 31].

Comparing the ${R}^{2}$ statistics only (Fig. 2), we found that Minimac4 outperformed FImpute3 for the most common variants but not for the rarest variants. It is worth pointing out, that in sequence data, there is a large number of rare variants, and it has been suggested that many causal mutations for complex traits may be present at low frequency [32, 33]. Our result that FImpute3 performs better for rare variants corroborates the results of Ma et al. [34] who reported a higher accuracy for rare variants with FImpute for imputation from 54 to 777 K in comparison to Beagle, Impute2, findhap, and AlphaImpute. In addition, Sargolzaei et al. [19] found that FImpute was able to call low-frequency variants with higher accuracy than Beagle and Impute2. According to [19], the observed advantage of FImpute for imputing SNPs with a low MAF could be due to the fact that most rare variants are recent and located within long haplotypes, which are quite efficiently exploited by the FImpute imputation algorithm. It is worth mentioning that our findings do not agree with those of Pausch et al. [7] who reported higher imputation accuracies for low-frequency variants with Minimac3 than with FImpute2 in dairy cattle.

On the one hand, the FImpute algorithm starts the phasing and imputation processes by exploiting the close relationships between individuals and by searching for the longest shared haplotypes that usually have a lower frequency in the population. By using an overlapping sliding window (OSW) approach, FImpute captures first the more accurate information from the close relatives by moving long windows along a chromosome and then exploits information from more distant relationships by gradually shrinking the window size in each chromosomal sweep [19]. Essentially, the longer is the shared haplotype (close relatedness), the more accurate is the imputation [35], which makes FImpute quite efficient in imputing rare variants even without pedigree information [19]. On the other hand, Minimac implements an algorithm based on a probabilistic model using a hidden Markov method that exploits similarities between haplotypes in small genomic segments [36]. In this case, to accurately impute rare variants, a large reference population is needed. Das et al. [36] reported that the imputation quality of sequenced rare variants using Minimac3 increased from ${R}^{2}$ = 45.3% to ${R}^{2}$ = 77.2% by increasing the reference panel from 1092 to 32,390 animals.

In terms of computational performance, FImpute is known to be an extremely fast software. In their study [7], Pausch et al. have run Eagle v2.3 and Minimac3 on 10 processors per chromosome and FImpute v2.2 on a single processor and found that the computing costs to impute sequence variants using Eagle-Minimac were more than ten times higher than using FImpute. In our study, parallelizing the imputation of the 29 bovine autosomes using 58 processors, FImpute3 took around 18 min for phasing and imputing each run of the fivefold imputation analysis, whereas the Eagle-Minimac4 approach took approximately 7.4 h (6.7 h for phasing using Eagle; 40 min to convert VCF to M3VCF using Minimac3; and 55 s for imputing with Minimac4).

Achieving high imputation accuracy is crucial for an effective use of imputed sequence genotypes in genetic evaluations of a specific population and the first priority is to choose a group of animals for which the number of haplotypes present in the reference population is maximized [4]. Efficient computational approaches in terms of accuracy and speed are also of relevance due to the challenge of imputing millions of SNPs, many with low-frequency minor alleles that are more difficult to accurately impute. In our application on beef cattle, we imputed whole-genome sequence variants with high accuracy with a relatively small reference group, which suggests that many of the haplotypes in the Nellore breed are captured in the group of influential Nellore sires selected for sequencing.

Regardless of the imputation approach, quality control of pre-imputation genotypes plays an important role for reaching a high imputation accuracy of sequenced variants. As shown in [Additional file 3: Figure S3], the imputation efficiency of both FImpute3 and Minimac4 decreased under a less strict genotype filtering scenario (same quality control procedure as described in the Methods section, except that the phred-scaled confidence score was not used). [Additional file 3: Figure S3] also shows that FImpute3 results were more affected by such a less conservative genotype filtering than Minimac4, given that we observed no difference in the imputation of rare variants between software and that the Minimac4 superiority for the most common variants was consistently higher. Slightly higher overall accuracies of imputed sequencing genotypes have been reported in cattle [7] and sheep [25] by combining Eagle and Minimac in comparison to FImpute. It should be noted that the FImpute algorithm is designed for high-quality genotypes that are obtained from DNA array technology [19]. Therefore, more strict quality checks on input genotypes driven from next-generation sequence data can be very effective in increasing imputation accuracy of FImpute3 [see Additional file 3: Figure S3]. The approach using Eagle and Minimac was less sensitive to the pre-imputation genotype quality check than FImpute.

As in [7, 25], average imputation accuracies were computed for successive 1-Mb windows across each chromosome to identify possible intra-chromosomal poorly imputed regions. Although high imputation accuracies have been achieved across the genome, some genomic regions presented a pronounced decrease in average accuracies [see Additional file 4: Figure S4]. Such intrinsically hard-to-impute genomic regions using sequencing data have been reported in humans [37], cattle [7], and sheep [25]. Their existence could be related with polymorphism and heterozygosity level, GC content, segmental duplications, assembly errors, and density of HD and sequencing variants [5, 7, 25, 37].

In Fleckvieh cattle, Pausch et al. [7] detected segments with high imputation errors on chromosomes 5, 10, 12, 15, and 23 at positions where the bovine genome contains large segmental duplications. Interestingly, in our study we detected the same hard-to-impute segments reported in [7] but with higher imputation accuracies. For instance, according to [7] the regions between 70 and 77 Mb on chromosome 12 and between 25 and 30 Mb on chromosome 23 could not be imputed using FImpute2 and were wrongly imputed using Minimac3 due to the presence of large segmental duplications associated with a low HD SNP coverage and high sequence variant density. Here, these regions were imputed with a moderate average accuracy using both FImpute3 and Minimac4, except the position 73 Mb on chromosome 12 that was imputed with a low accuracy [see Additional file 4: Figure S4]. Such a better imputation at consistently hard-to-impute genomic regions could be due to the use of the newest and improved reference genome assembly (ARS-UCD1.2) in our study. By providing a significant improvement in per-base accuracy over previous cattle assemblies [38], using the ARS-UCD1.2 genome assembly for aligning and variant calling might contribute to an overall higher imputation accuracy across the genome including regions that are intrinsically difficult to accurately impute. In addition, as observed in [7, 25], intra-chromosomal imputed segments with low accuracy often present low HD SNP coverage and high sequence variant density [see Additional file 5 Figure S5]. However, in contrast to these studies, we did not observe such a higher than usual density of sequence variants at these lower imputed regions [see Additional file 5: Figure S5], which could also be related to the use of an improved reference genome assembly.

**Fig. 3:**
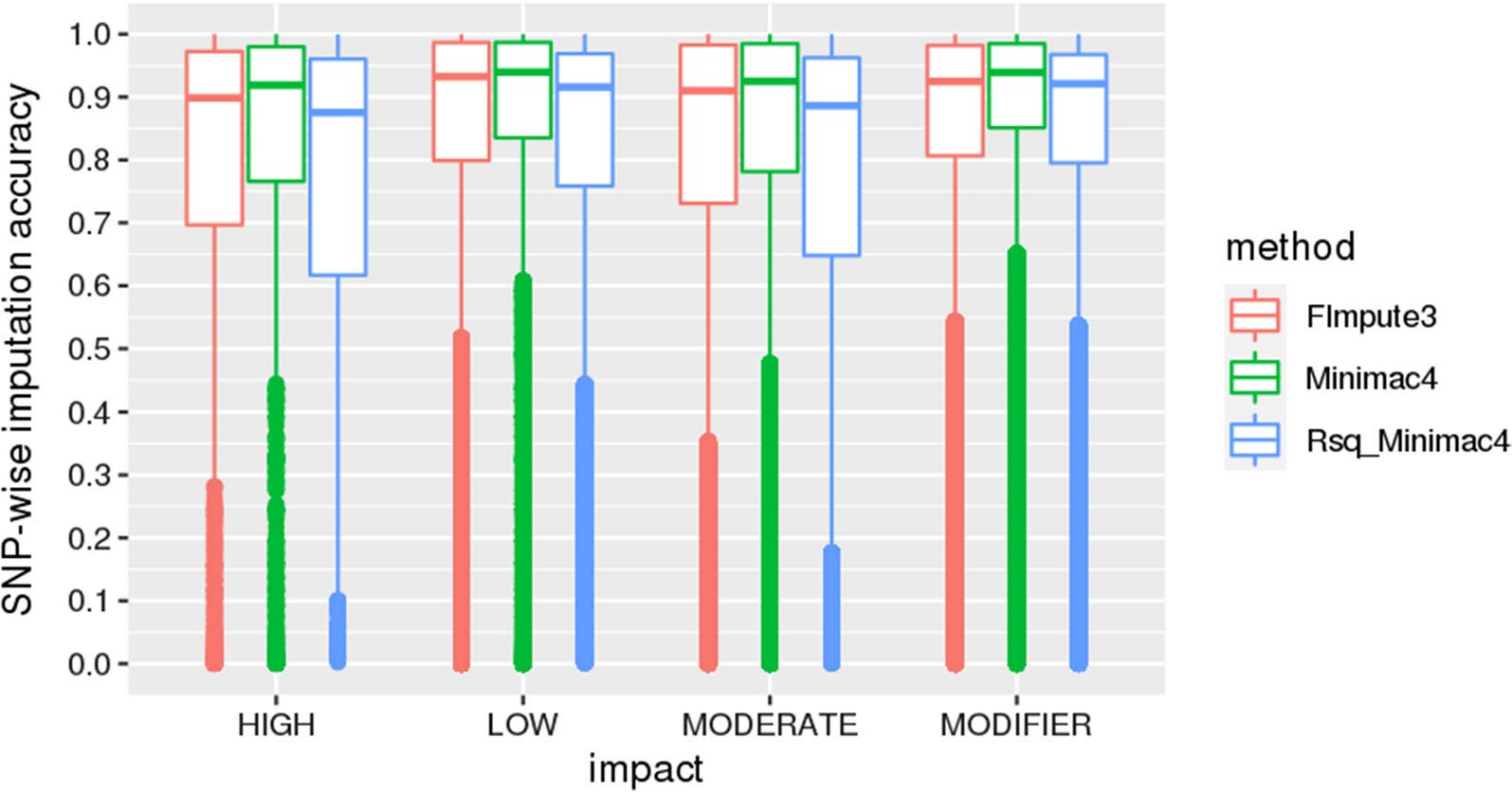
SNP-wise imputation accuracy by annotation class. High: variants that cause premature stop codons, loss of function or trigger nonsense-mediated decay; Low: variants that are mostly harmless or unlikely to change protein behavior; Moderate: non-disruptive variants that might change protein effectiveness; Modifier: non-coding variants or variants that affect non-coding genes, for which predictions are difficult or there is no evidence of impact

Figure 3 displays the imputation accuracy of whole-genome sequence genotypes by classes of annotation. The imputed variants were grouped according to their impact (high, moderate, low, and modifier) in transcripts. Variants with a ‘high’ impact include splice acceptor and splice donor, start- and stop-lost, and stop-gained variants. The missense variants are grouped into the ‘moderate’ class. ‘Low’ impact variants include synonymous, stop retained and splice region variants. Intergenic, intronic, up- and down-stream, and UTR variants are grouped as ‘modifier’. Additional file 6: Figure S6 shows the imputation accuracy for the variants from the ‘modifier’ group divided by classes, in addition to the missense variants.

The observed high median with a relatively low interquartile range (Fig. 3) highlighted that high imputation accuracies were achieved by using both FImpute3 and Minimac4 for all classes of annotation. Interestingly, the Minimac4 Rsq statistic exhibited a larger dispersion compared to the empirical imputation accuracies, especially for the variants of high and moderate impact. The imputation accuracy of variants into these two functional classes is especially relevant since they incorporate variants that may directly influence the expression of phenotypes. However, as they usually include a high proportion of low-frequency mutations, these types of variants may be more difficult to impute accurately [25]. Indeed, approximately 15% of the variants from the ‘high’ and ‘moderate’ functional classes present MAF ≤ 0.03 and, as shown in Table 3, there was an overall trend for increased average accuracy moving from high to low impact variants. Taking only the low-frequency variants into account, higher imputation accuracies were achieved using FImpute3 for all classes of annotation, compared with Minimac4 (Table 3).

**Table 3:** SNP-wise imputation accuracy from the Bovine HD BeadChip (~ 777 K) to whole-genome sequence in Nellore cattle using FImpute3 and Minimac4 by classes of MAF and functional annotation

	Min	1st Qu	Median	Mean	3rd Qu	Max
*High*						
Overall						
FImpute3	0.00	0.70	0.90	0.78	0.97	1.00
Minimac4	0.00	0.76	0.92	0.79	0.98	1.00
Rsq_Minimac4	0.00	0.62	0.87	0.75	0.96	1.00
MAF ≤ 0.03						
FImpute3	0.00	0.49	0.79	0.69	1.00	1.00
Minimac4	0.00	0.35	0.74	0.65	1.00	1.00
Rsq_Minimac4	0.11	0.34	0.48	0.49	0.63	1.00
*Moderate*						
Overall						
FImpute3	0.00	0.73	0.91	0.80	0.98	1.00
Minimac4	0.00	0.78	0.92	0.81	0.98	1.00
Rsq_Minimac4	0.00	0.65	0.87	0.77	0.96	1.00
MAF ≤ 0.03						
FImpute3	0.00	0.56	0.83	0.74	1.00	1.00
Minimac4	0.00	0.49	0.74	0.69	1.00	1.00
Rsq_Minimac4	0.03	0.35	0.50	0.50	0.65	1.00
*Low*						
Overall						
FImpute3	0.00	0.80	0.93	0.85	0.99	1.00
Minimac4	0.00	0.83	0.94	0.86	0.99	1.00
Rsq_Minimac4	0.00	0.76	0.91	0.81	0.97	1.00
MAF ≤ 0.03						
FImpute3	0.00	0.66	0.85	0.78	1.00	1.00
Minimac4	0.00	0.50	0.79	0.71	1.00	1.00
Rsq_Minimac4	0.02	0.37	0.52	0.52	0.66	1.00
*Modifier*						
Overall						
FImpute3	0.00	0.81	0.92	0.86	0.98	1.00
Minimac4	0.00	0.85	0.94	0.88	0.98	1.00
Rsq_Minimac4	0.00	0.79	0.92	0.84	0.97	1.00
MAF ≤ 0.03						
FImpute3	0.00	0.61	0.83	0.75	1.00	1.00
Minimac4	0.00	0.49	0.74	0.70	1.00	1.00
Rsq_Minimac4	0.00	0.37	0.52	0.52	0.66	1.00

*High*, variants that cause premature stop codons, loss of function or trigger nonsense-mediated decay; *low*, variants mostly harmless or unlikely to change protein behavior; *moderate*, non-disruptive variants that might change protein effectiveness; *modifier*, non-coding variants or variants affecting non-coding genes, where predictions are difficult or there is no evidence of impact

As shown by Druet et al. [4], compared to SNP arrays, the use of sequenced variants can significantly increase the prediction accuracy in genomic evaluation when the QTL has a low MAF. Therefore, non-synonymous polymorphisms such as missense variants are of paramount importance since they are more likely associated with complex traits in cattle [39] and, usually, present a high proportion of low-frequency variants which are more difficult to impute accurately [25]. However, in practice, most of the total genetic variation for complex traits is explained by the common sequence variants [33]. Thus, our results indicate that, for GWAS, two separate whole genome searches on imputed genotypes from FImpute3 and Minimac4 could be complementary with regard to rare and common variants. For routine genomic evaluation where the overall accuracy per animal and computing efficiency are more important, FImpute3 might have an advantage.

## Conclusions

High imputation accuracy to whole-genome sequence was achieved in Nellore beef cattle. In general, common variants were imputed with higher accuracy by using Eagle-Minimac4, but, in terms of computational efficiency and higher imputation accuracy for low-frequency variants, there were advantages in using FImpute3.

## Supplementary Information

**Additional file 1: Figure S1.** Principal component analysis based on genomic relationship matrix showing the genetic structure of the sequenced sires (in red) relative to our Nellore reference population of about 10.000 genotyped animals.

**Additional file 2: Figure S2.** Smoothed conditional means (see http://search.r-project.org/library/ggplot2/html/geom_smooth.html) of the SNP-wise imputation accuracies by minor allele frequency (MAF). ${R}^{2}$: Squared Pearson’s correlation between observed and imputed genotypes; PERC: percentage of genotypes correctly imputed.

**Additional file 3: Figure S3.** Smoothed conditional means (see http://search.r-project.org/library/ggplot2/html/geom_smooth.html) of the SNP-wise imputation accuracies by minor allele frequency (MAF), comparing analyses using a more or less strict genotype filtering before imputations. FImpute3 and Minimac4 methods correspond to the squared Pearson’s correlation between observed and imputed genotypes; and Rsq_Minimac4 method is an estimate of the squared correlation between imputed genotypes and true, unobserved genotypes (see https://genome.sph.umich.edu/wiki/Minimac3_Info_File).

**Additional file 4: Figure S4.** SNP-wise imputation accuracies by successive 1-Mb windows across all autosomes. FImpute3 and Minimac4 methods correspond to the squared Pearson’s correlation between observed and imputed genotypes; and Rsq_Minimac4 method is an estimate of the squared correlation between imputed genotypes and true, unobserved genotypes (see https://genome.sph.umich.edu/wiki/Minimac3_Info_File).

**Additional file 5: Figure S5.** Number of variants in the high-density (HD) SNP panel and in the whole-genome sequencing (WGS) reference panel per successive 1-Mb windows across all autosomes. The red line represents the number of SNPs per Mb included in HD and the blue line represents the number of SNPs (× 100) included in WGS.

**Additional file 6: Figure S6.** SNP-wise imputation accuracy for the intergenic, intronic, missense, up- and down-stream, and UTR variants.

## Data Availability

The data used in this study were obtained under license and thus cannot be made publicly available.
