# Supplementary figures and images for: Imputation accuracy to whole-genome sequence in Nellore cattle
Source: Genet Sel Evol. 2021 Mar 12;53:27. doi: 10.1186/s12711-021-00622-5 (PMC7953568; doi:10.1186/s12711-021-00622-5)

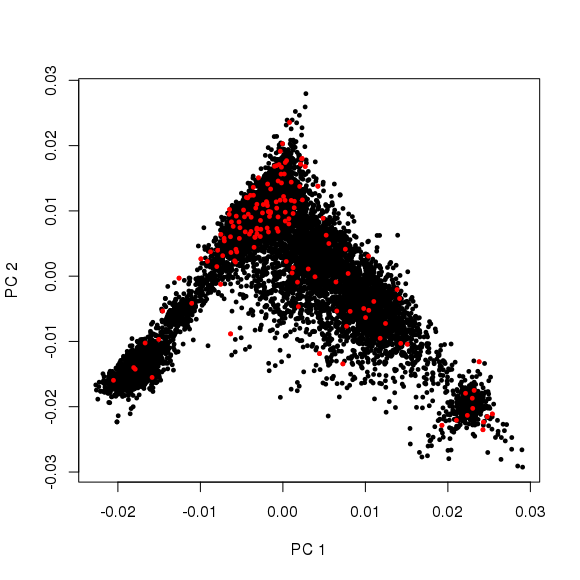

Supplement: Supplementary file 1 — Additional file 1: Figure S1. Principal component analysis based on genomic relationship matrix showing the genetic structure of the sequenced sires (in red) relative to our Nellore reference population of about 10.000 genotyped animals. [file 12711_2021_622_MOESM1_ESM.tif]

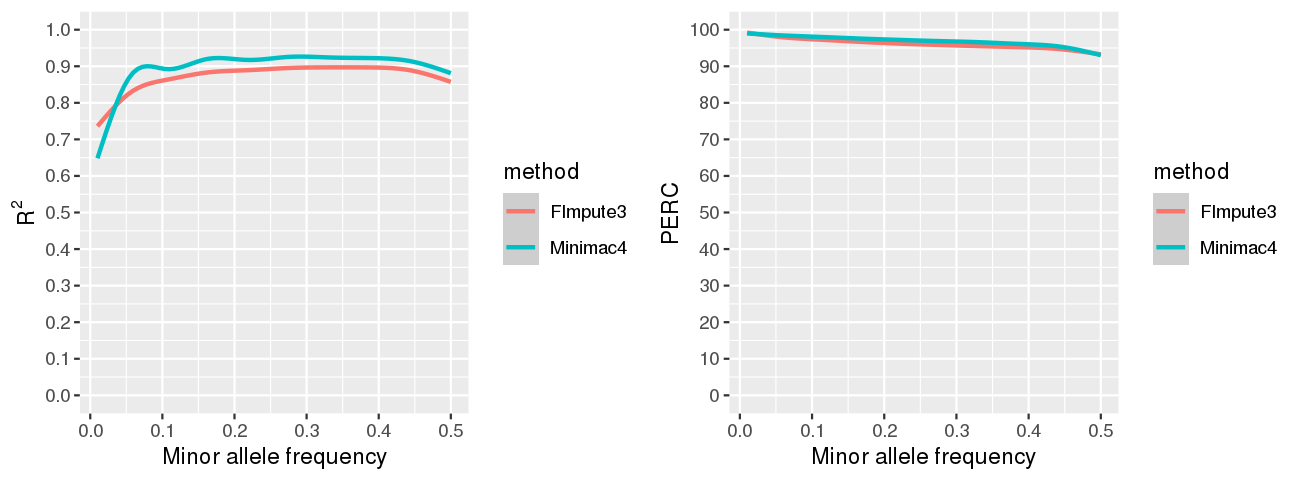

Supplement: Supplementary file 2 — Additional file 2: Figure S2. Smoothed conditional means (see http://search.r-project.org/library/ggplot2/html/geom_smooth.html) of the SNP-wise imputation accuracies by minor allele frequency (MAF). ${R}^{2}$R2: Squared Pearson’s correlation between observed and imputed genotypes; PERC: percentage of genotypes correctly imputed. [file 12711_2021_622_MOESM2_ESM.png]

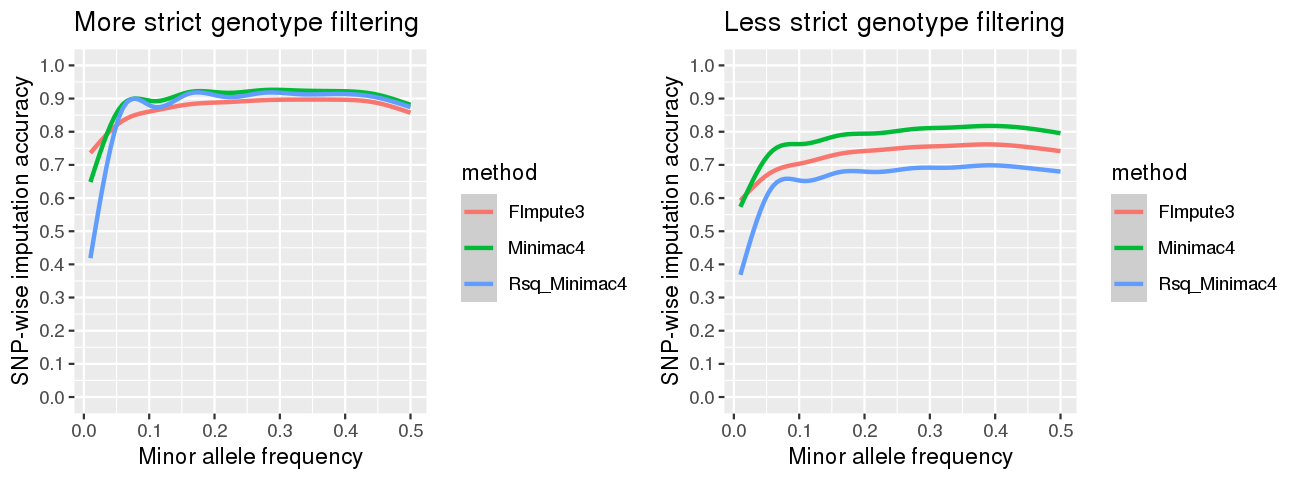

Supplement: Supplementary file 3 — Additional file 3: Figure S3. Smoothed conditional means (see http://search.r-project.org/library/ggplot2/html/geom_smooth.html) of the SNP-wise imputation accuracies by minor allele frequency (MAF), comparing analyses using a more or less strict genotype filtering before imputations. FImpute3 and Minimac4 methods correspond to the squared Pearson’s correlation between observed and imputed genotypes; and Rsq_Minimac4 method is an estimate of the squared correlation between imputed genotypes and true, unobserved genotypes (see https://genome.sph.umich.edu/wiki/Minimac3_Info_File). [file 12711_2021_622_MOESM3_ESM.png]

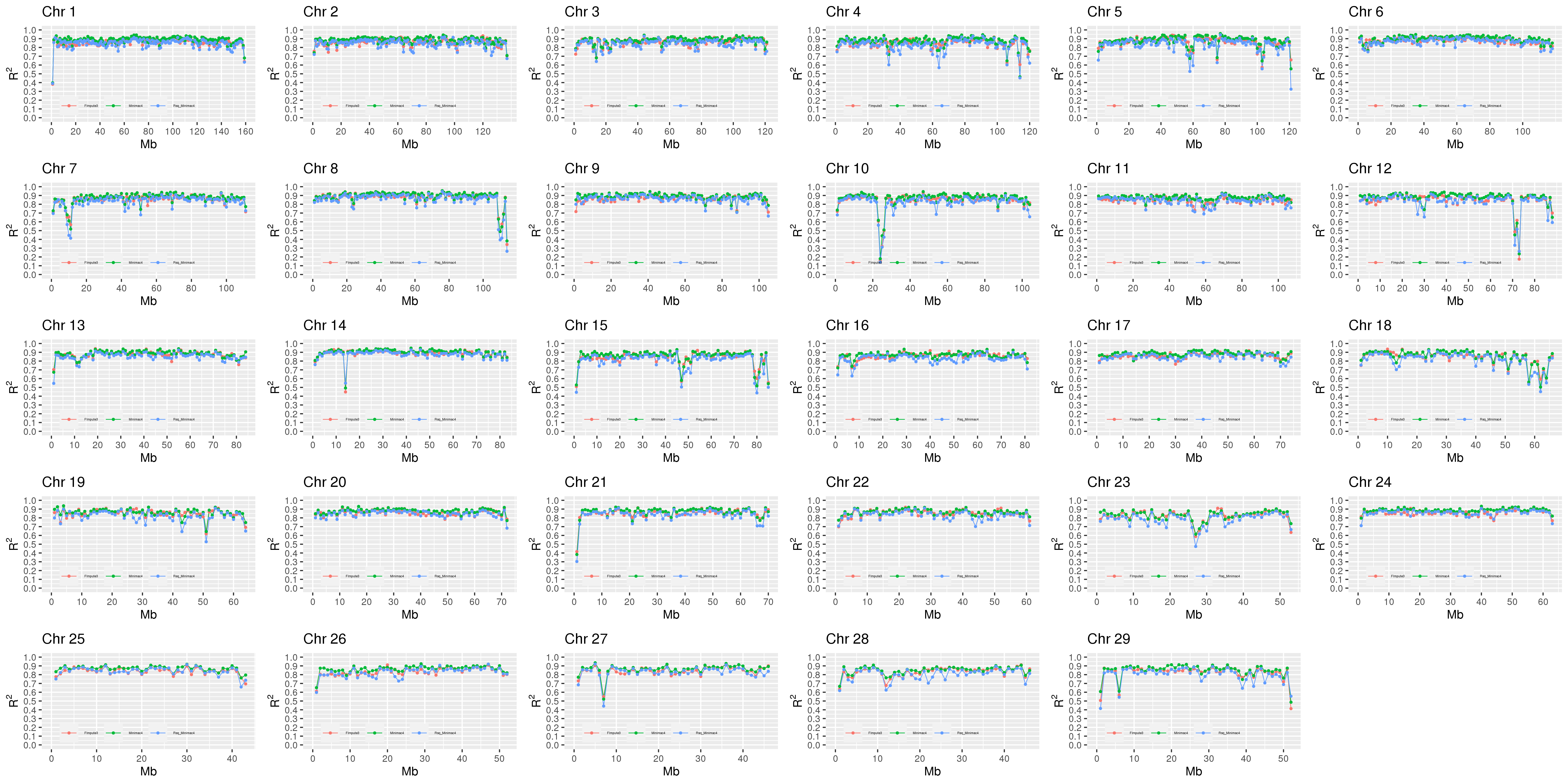

Supplement: Supplementary file 4 — Additional file 4: Figure S4. SNP-wise imputation accuracies by successive 1-Mb windows across all autosomes. FImpute3 and Minimac4 methods correspond to the squared Pearson’s correlation between observed and imputed genotypes; and Rsq_Minimac4 method is an estimate of the squared correlation between imputed genotypes and true, unobserved genotypes (see https://genome.sph.umich.edu/wiki/Minimac3_Info_File). [file 12711_2021_622_MOESM4_ESM.png]

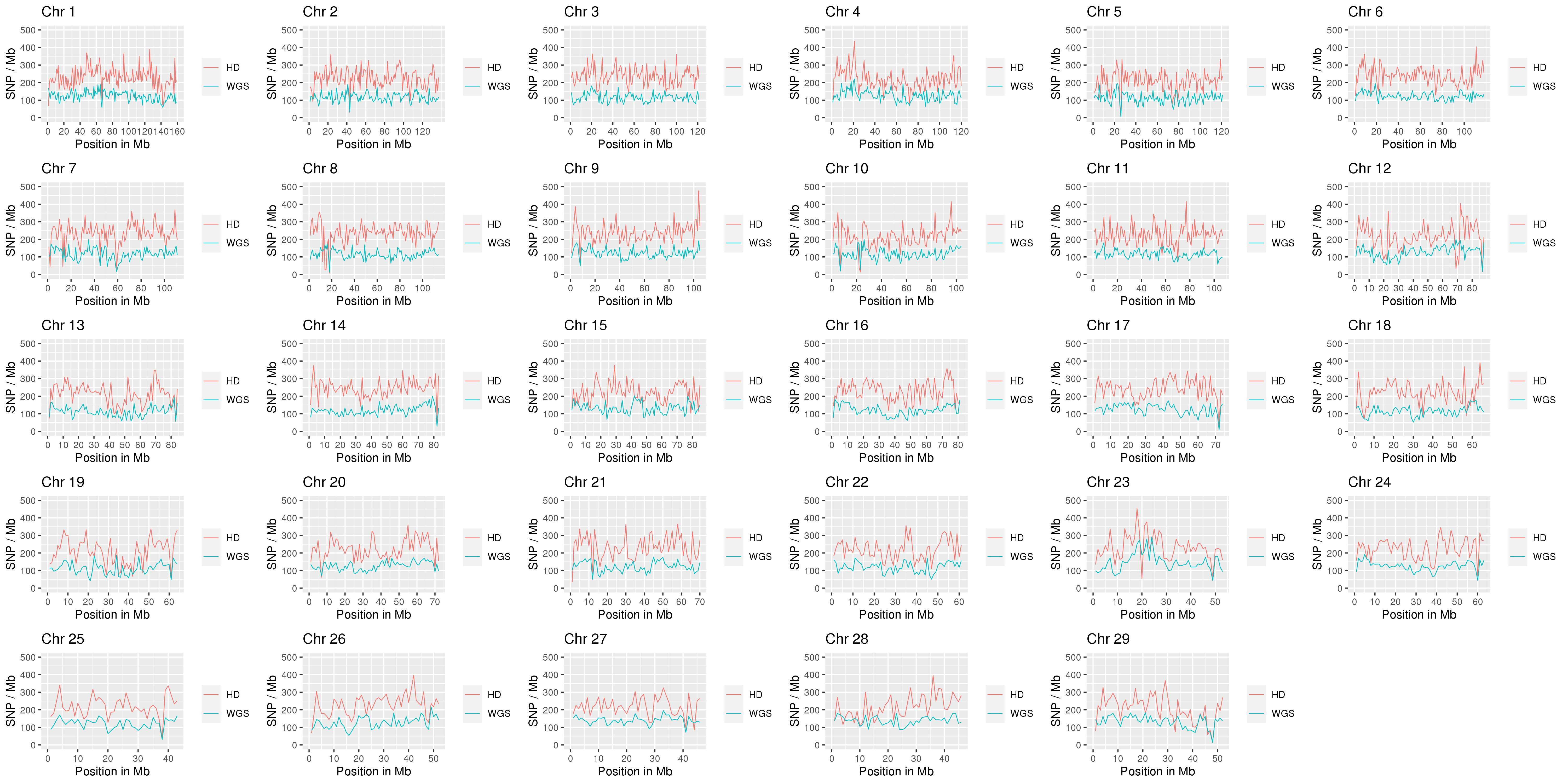

Supplement: Supplementary file 5 — Additional file 5: Figure S5. Number of variants in the high-density (HD) SNP panel and in the whole-genome sequencing (WGS) reference panel per successive 1-Mb windows across all autosomes. The red line represents the number of SNPs per Mb included in HD and the blue line represents the number of SNPs (× 100) included in WGS. [file 12711_2021_622_MOESM5_ESM.png]

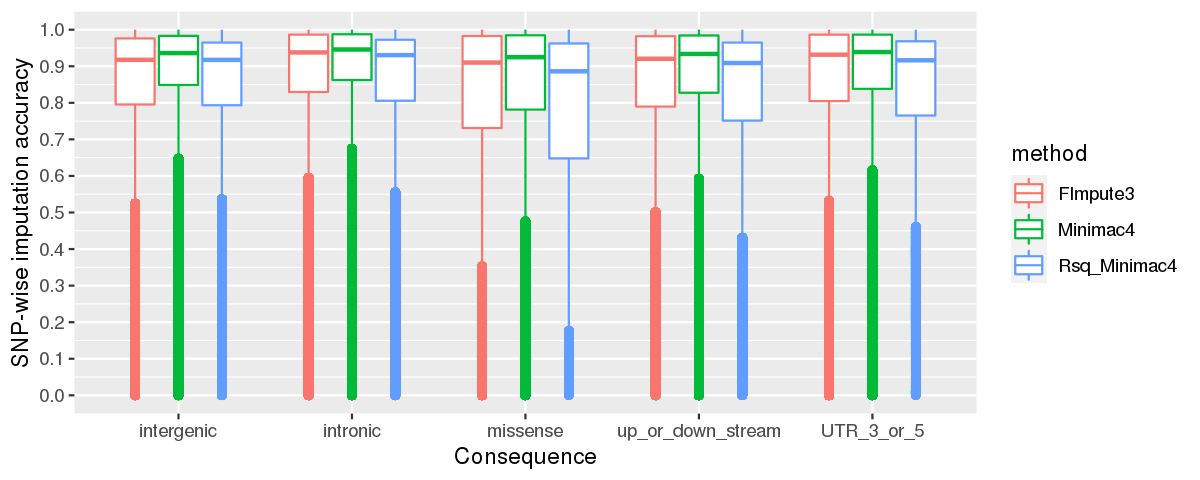

Supplement: Supplementary file 6 — Additional file 6: Figure S6. SNP-wise imputation accuracy for the intergenic, intronic, missense, up- and down-stream, and UTR variants. [file 12711_2021_622_MOESM6_ESM.png]
